# Male cancer patient sperm cryopreservation for fertility preservation: 10-year monocentric experience

**DOI:** 10.1186/s12610-021-00140-w

**Published:** 2021-09-16

**Authors:** Xiao Liu, Bo Liu, Shasha Liu, Yang Xian, Wenrui Zhao, Bin Zhou, Xiao Xiao, Li Wang, Xiaofang Zhu, Bizhen Shu, Min Jiang, Fuping Li

**Affiliations:** 1 Human Sperm Bank, Key Laboratory of Birth Defects and Related Diseases of Women and Children, West China Second University Hospital, Sichuan University, Ministry of Education, Sichuan University, 610041 Sichuan, P.R. China; 2 Laboratory of Molecular Translational Medicine, Center for Translational Medicine, Key Laboratory of Birth Defects and Related Diseases of Women and Children, Clinical Research Center for Birth Defects of Sichuan Province, West China Second University Hospital, Sichuan University, Ministry of Education, Sichuan University, 610041 Chengdu, Sichuan P.R. China; 3 Human Sperm Bank, West China Second University Hospital, Sichuan University, No. 1416, Section 1, Chenglong Avenue, Chengdu, China

**Keywords:** sperm cryopreservation, male infertility, oncology, semen quality, assisted reproductive technologies, Cryoconservation des spermatozoïdes, infertilité masculine, oncologie, qualité du sperme, Assistance Médicale à la Procréation

## Abstract

**Background:**

Sperm cryopreservation, an effective method for preserving male fertility, is very advantageous for men suffering from cancer. Unfortunately, as both physicians and cancer patients are unaware of the possibilities for sperm cryopreservation, the data on evaluation of semen parameters and disposition of cryopreserved samples among Chinese cancer patients are scarce.

**Results:**

Male tumours were classified into six major types, germ cell tumours (26 %), haematological neoplasms (28 %), head and neck cancers (19 %), thoracic tumours (4 %), abdominal tumours (10 %), and others (13 %). Haematological neoplasm was the most prevalent cancer among our cohort of patients who opted for sperm banking, followed by germ cell tumours. Patients with germ cell tumours had the lowest pre-thaw and post-thaw seminal sperm concentrations. We separately compared patients with testicular tumours, lymphoma, and leukaemia, and found that leukaemia patients had the lowest pre-thaw sperm concentrations. Most cancer patients (58 %) chose to keep their specimens stored, while 31 % chose to discard the specimens. Over the years, only 13 patients (4 %) returned to use their spermatozoa by assisted reproductive technology. Of the stored samples, patients with germ cell tumours constituted the highest proportion (29.3 %). Moreover, the percentage of haematological neoplasm patients who had no spermatozoa frozen was the highest (46.2 %).

**Conclusions:**

The present data confirm the deleterious impact of various cancers on semen quality. Leukaemia was associated with the worst semen quality and the highest number of semen samples that could not be frozen. We suggest that sperm quality may have decreased even before anti-neoplastic treatment and that sperm banking before treatment should be strongly recommended for cancer patients. A sperm banking programme before gonadotoxic therapy requires close cooperation between assisted reproduction centres and cancer clinics.

## Résumé

**Contexte:**

Lacryoconservation des spermatozoïdes, méthode efficace pour préserver lafertilité masculine, est très avantageuse pour les hommes atteints de cancer.Malheureusement, comme les médecins et les patients atteints de cancersignorent les possibilités de cryoconservation du sperme, les données surl’évaluation des paramètres spermatiques et le devenir des échantillonscryopréservés parmi les hommes chinois atteints de cancers sont rares.

**Résultats:**

Lestumeurs masculines ont été classées en six types principaux, tumeurs germinales(26%), néoplasmes hématologiques (28%), cancers de la tête et du cou (19%),tumeurs thoraciques (4%), les tumeurs abdominales (10%), et les autres (13%).Les néoplasmes hématologiques étaient le cancer le plus répandu parmi notrecohorte de patients qui ont opté pour la mise en banque de leur sperme, suivisdes tumeurs à cellules germinales. Les patients atteints de tumeurs germinalesprésentaient les concentrations de spermatozoïdes pré-congélation etpost-décongélation les plus faibles. Nous avons comparé séparément les patientsatteints de tumeurs testiculaires, de lymphome, et de leucémie, et avonsconstaté que ceux atteins de leucémie présentaient les plus bassesconcentrations de spermatozoïdes pré-congélation. Une majorité de patientsatteints de cancer (58 %) ont choisi de conserver en banque leurs échantillonscongelés, tandis que 31 % ont choisi de jeter leurs échantillons congelés. Aufil des ans, seuls 13 patients (4%) sont revenus pour utiliser leurs spermatozoïdespar Assistance Médicale à la Procréation. Parmi les échantillons mis en banque,ceux des patients atteints de tumeurs germinales constituaient la proportion laplus élevée (29,3 %). Par ailleurs, les patients atteins de cancershématologiques constituaient le pourcentage le plus élevé (46.2%) de patientsqui n’avaient eu aucun spermatozoïde congelé.

**Conclusions:**

Lesdonnées rapportées confirment l’impact délétère des divers cancers sur laqualité du sperme. Les leucémies étaientassociées à la pire qualité du sperme et au plus grand nombre d’échantillons desperme qui ne pouvaient pas être congelés. Nous pensons que la qualité desperme pourrait avoir diminué avant même le traitement antinéoplasique, et quela mise en banque de sperme avant le traitement devrait être fortementrecommandée pour les patients atteints de cancers. Un programme de mise enbanque du sperme avant traitement gonadotoxique nécessite une étroitecoopération entre les centres d’Assistance Médicale à la Procréation et les cliniquesde cancérologie.

## Introduction

According to the World Health Organization (WHO), infertility will become the third-most prevalent disease in the 21st century after cancer and cardiovascular diseases, affecting human life and health [1]. Studies have reported that the distress related to infertility is more prevalent among male cancer survivors than among unaffected men [2–4]. The increase in the number of cancer survivors has highlighted the need for long-term improvement in their quality of life and the demand for reproductive physicians, especially with respect to cancer-related infertility [5, 6]. Therefore, studies have investigated the potential negative effects of cancer therapies, including chemotherapy and radiotherapy, and malignancy itself on male fertility [7]. Sperm banking for male cancer patients is considered as the most effective method for preserving fertility [8, 9]. Several studies have been reported that testicular cancer and haematological diseases affect male fertility, but many other cancers including head and neck tumor and sarcoma that were little reported about male fertility.

In China, only a few reports have been published on fertility preservation in male cancer patients [10]. One of the studies conducted a retrospective review of sperm cryopreservation for 143 male cancer patients at the Human Sperm Bank of the National Research Institute for Family Planning in Beijing [11].

Given the late start of this programme in China, there is not much awareness about human fertility preservation, highlighting the need to develop a systemic and patient-centric programme for offering cryopreservation services to all male cancer patients. The Human Sperm Bank, West China Second University Hospital, Sichuan University, has been offering sperm banking to patients for 10 years. In comparison with other sperm banks in China, our unit has access to a greater number of subjects and has received 1039 sperm samples for cryopreservation for birth demand. Overall, 339 cancer patients banked their spermatozoa for fertility preservation. To our knowledge, we have accessed most male cancer patients who came to bank their spermatozoa for fertility conservation in southwest China.

This is a 10-year retrospective study of a population of patients referred for sperm cryopreservation before cancer therapy. The primary aim of our study was to evaluate semen parameters and the disposition of cryopreserved samples among different cancer patients in our 10-year experience with sperm banking. We simultaneously focused on reproductive outcomes using cryopreserved semen via assisted reproductive technology (ART).

## Materials and methods

### Patients

In accordance with the guidelines and processes established by our sperm bank, all cancer patients seeking fertility preservation were counselled by an andrology physician and fully informed about the procedure, including the process entailed, costs, future use and storage duration. After receiving written and oral information, men were required to sign a cryostorage consent form that stated that spermatozoa would be discarded upon loss to follow-up and specified how the spermatozoa were to be used. It was stipulated that the stored gametes were only to be used for married couples. Moreover, no use in posthumous conception was allowed. Sperm cryopreservation was performed according to a standardised protocol in the sperm bank and only on semen samples containing motile spermatozoa. If no motile spermtozoa were detected, the findings were discussed with the patient and the sample was not cryopreserved. The disposition categories were continued storage within our unit, discarded, failure to bank, used, and patient’s death. The type of cancer was determined from the oncologist’s letter or by histological diagnosis. We followed up cancer patients by telephone.

### Semen samples

Semen samples were obtained by masturbation in sterile containers, which were placed in an incubator at 37 °C until liquefaction and analysed within 1 h. The following semen parameters were evaluated according to the WHO 1999, 2010 guidelines: sperm concentration, volume, progressive motility (grades a + b), and morphology [12, 13]. Sperm motility and concentration were detected using a Suiplus semen analysis (SSA) automatic detection system, which consists of a phase-contrast microscope (OLYMPUS CX41), a micro camera (Basler acA780—75gc), SSA software system (Beijing SuiJia Software Co., Ltd, China) [14] and a Makler counting chamber (Sefi Medical Instruments, Haifa, Israel).

Sperm cryopreservation was performed as follows. The liquefied semen sample was transferred to a 2.0 mL sterile cryotube with Sperm Cryoprotectant Kit (Anhui Anke Biotechnology (Group) Co.,LTD, China, glycerol-egg yolk free cryopreservative medium, final glycerol concentration of 7.5 % [15, 16]) added dropwise until a 3:1 sample: medium ratio. The final volume of diluted seminal fluid in a tube was 1mL.

The straws were stored at 4 °C for 15 min and then suspended 5 cm above of liquid nitrogen for 10 min before being stored in liquid nitrogen. For semen thawing, straws were placed at room temperature for 1 min and then transferred to a beaker at 37 °C until all ice crystals had melted. Azoospermia was confirmed by centrifugation of the entire semen sample at 3000 ×*g* for 15 min.

### Statistical analysis

The statistical methodology comprised one-way analysis of variance and Kruskal-Wallis tests. In all cases, statistical significance was set at *p* < 0.05.

## Results

### Study population

**Fig. 1:**
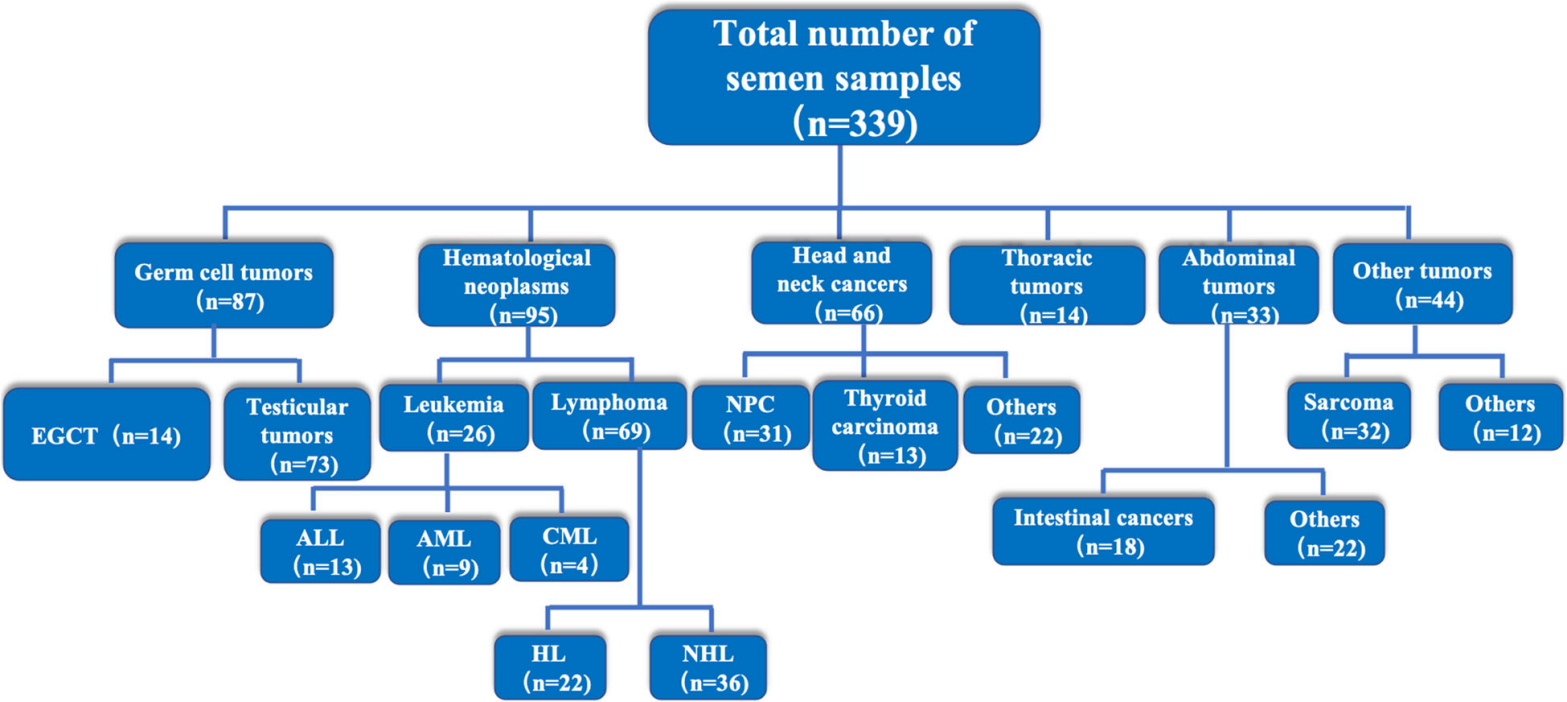
Flow diagram of male cancer patients who presented to Sichuan Human Sperm Bank from 2010 to 2019 for sperm cryopreservation

**Fig. 2:**
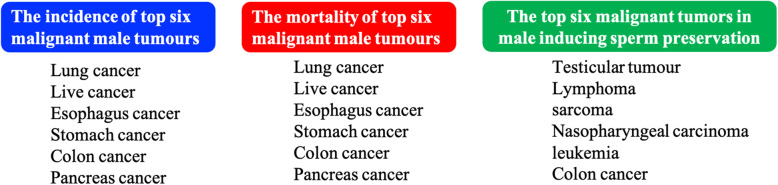
The incidence of male, mortality of male and the top six malignant tumors in male inducing sperm preservation in Sichuan

A total of 339 male cancer patients who opted for sperm cryopreservation were recruited from January 2010 to December 2019 (Fig. 1). The type of cancers among these 339 patients included germ cell tumours (26 %), haematological neoplasms (28 %), head and neck cancers (19 %), thoracic tumours (4 %), abdominal tumours (10 %), and others (13 %). Germ cell tumours included testicular tumours and extragonadal germ cell tumours. Haematological neoplasms comprised lymphoma and leukaemia. The “other” group was composed of skin, sarcoma, and peripheral Schwannoma. The cancer incidence rate for men in Sichuan Province is 303.60 per 100,000. The six more common cancers among men (based on incidence) were lung cancer (74.26 per 100,000), liver cancer (48.3 per 100,000), oesophageal cancer (42.62 per 100,000), gastric cancer (37.55 per 100,000), colorectal cancer (28.58 per 100,000), and pancreatic cancer (7.38 per 100,000) [17]. We compared the incidence, mortality, and sperm preservation among patients with top six tumour types in Sichuan using the data from the Sichuan cancer registries. Only colon cancer was in the top six tumour types for sperm preservation (Fig. 2). Therefore, the high incidence of tumours may not be related to high sperm preservation rate, owing to high mortality. The types of tumours that preserve fertility are those affected by reproduction or have a younger incidence. In our unit, we found not only testicular tumours but also extragonadal germ cell tumours among men with the need for fertility preservation. The age of the patients was 26.7 ± 6.8 years, and 31 patients (9.9 %) were adolescents (below 18 years of age).

### Semen parameters

Most samples were collected in sterile containers by masturbation after an abstinence period of 2–7 days. Some patients had sexual abstinence period of longer than 1 week, owing to emergency treatment such as needing radiation or chemotherapy (Tables 1 and 2). The mean semen volume was 3.4 ± 1.5 mL, and the median number of straws stored was 4 per patient among different cancer groups. Before cryopreservation, the overall median sperm concentration was 47 × 10^6^/mL and the progressive motility was 49 %. Post-thaw analyses after cryopreservation revealed a median concentration of 30 × 10^6^/mL and progressive motility of 28 %. No difference was observed in the change from pre-freeze to post-thaw progressive motility and the recovery rate between various cancer groups. The sperm pre-count, total sperm number, and post-thaw concentration were significantly lower in germ cell tumours than in other five cancers (Table 1). We compared the data from leukaemia, lymphoma, and testicular cancer patients, and found that the patients with leukaemia had the poorest sample quality, consistent with the lower pre-freeze count and pre-freeze total sperm number (Table 2).

**Table 1:** Semen parameters at banking stratified by cancer types

	Germ cell tumors(*n* = 81)	Hematological neoplasms(*n* = 83)	Head and neck cancers(*n* = 61)	Thoracic tumors(*n* = 14)	Abdominal tumors(*n* = 32)	Other Tumors(*n* = 42)	*p*-Value^c^
abstinence time(days) ^a^	4.5 ± 1.9(*n* = 62,77 %^f^)	4.1 ± 1.4(*n* = 61, 73 %^f^)	4.8 ± 2.2(*n* = 53, 87 %^f^)	4.8 ± 2.1(*n* = 11,79 %^f^)	4.4 ± 1.8(*n* = 24,75 %^f^)	4.9 ± 2.0(*n* = 30,71 %^f^)	0.7262^d^
volume at banking (mL) ^a^	3.7 ± 1.5	3.2 ± 1.3	3.3 ± 1.6	3.8 ± 1.3	2.7 ± 1.3	3.5 ± 1.6	<0.0001 ^d^
numbers of samples banked ^b^	4(1–17)	4(1–10)	4(1–20)	4(2–5)	4(1–12)	4(1–8)	0.5431^e^
pre-count (10^6^/mL) ^b^	26(0.9–159)	41(0.7–222)	64(0.4–620)	47.5(0.4–108)	64(3.3–229)	54.5(1.2–226)	0.0015^e^
pre-total sperm number (10^6^) ^b^	81(3-726)	157(1-1701)	215(1-1612)	173(2-658)	209(12-1438)	149(4-710)	< 0.0001^e^
pre-progressive motility (%) ^b^	48.5(3.1–83)	48(1.04-75)	51.5(4–73)	53(18–79)	45(8–70)	50(9–81)	0.2334^e^
post-count (10^6^/mL) ^b^	16.5(0.6–89)	28(0.5–120)	42(3.7–193)	23(0.3–52)	40(2.2–120)	37(0.6–148)	0.0041^e^
post-progressive motility (%) ^b^	29(0.66-61)	29(2–60)	30.5(8–64)	23(6–55)	23(3–45)	30(0.7–66)	0.4099^e^
recovery rates (%) ^b^	58.8(2.2–89.6)	60(9.1–89.6)	62.4(12.1–89.3)	52.5(31.9–73.3)	59(15.2–80.4)	59.3(2.8–89.2)	0.8639^e^

a: mean ± standard deviation

b: median (range)

c: *p* < 0.05 significant

d: One-way analysis of variance among groups

e: Kruskal-Wallis tests was used among groups

f: the percent of abstinence time of the patients between 2 and 7 days as compared to all patients in each group pre-count, pre-total sperm number and pre-progressive motility: count of spermatozoa (million/ml), total sperm number (million/ejaculate) and percentage of spermatozoa with progressive motility before banking post-count, and post-progressive motility: count of spermatozoa (million/ml) and percentage of spermatozoa with progressive motility after thawing recovery rate (%): the percentage recovery of progressively motile spermatozoa after thawing as compared to fresh spermatozoa before banking

**Table 2:** Patients’ age and semen parameters of testicular tumor, leukemia and lymphoma

	Testicular tumors(*n* = 68)	Leukemia(*n* = 18)	Lymphoma(*n* = 65)	*p*-Value^c^
abstinence time(days) ^a^	4.4 ± 1.8(*n* = 50,74 %^f^)	4.7 ± 2.0(*n* = 12,67 %^f^)	4.1 ± 1.2(*n* = 55,85 %^f^)	0.7188^d^
age at banking (years)	27 ± 7.5	24.7 ± 7.2	25.1 ± 5.5	0.0115 ^d^
volume at banking (mL) ^a^	3.8 ± 7.8	2.8 ± 1.3	3.4 ± 1.3	0.0388^d^
pre-count (10^6^/mL) ^b^	30 (0.9–159)	18.5 (0.7–134)	48 (1.5–222)	0.0020^e^
pre-total sperm number (10^6^) ^b^	81(3-726)	52(1-1701)	172(2-1304)	0.0130^e^
pre-progressive motility (%) ^b^	48.5 (3.1–83)	41.5 (6.69-48)	50 (1.04-73)	0.1595^e^
post-count (10^6^/mL) ^b^	16.4 (0.6–89)	10.9 (0.5–92)	32 (1-120)	0.1436^e^
post-progressive motility (%) ^b^	28 (2.6–61)	18.5 (2–37)	30 (5–60)	0.0563^e^
Recovery rates (%) ^b^	59.5 (6.8–89.6)	53.7 (9.1–77)	60.8 (25–89)	0.5558^e^

a: mean ± standard deviation

b: median (range)

c: *p* < 0.05 significant

d: One-way analysis of variance among groups

e: Kruskal-Wallis tests was used among groups

f: the percent of abstinence time of the patients between 2 and 7 days as compared to all patients in each group

pre-count, pre-total sperm number and pre-progressive motility: count of spermatozoa (million/ml), total sperm number (million/ejaculate) and percentage of spermatozoa with progressive motility before banking post-count, and post-progressive motility: count of spermatozoa (million/ml) and percentage of spermatozoa with progressive motility after thawing recovery rate (%): the percentage recovery of progressively motile spermatozoa after thawing as compared to fresh spermatozoa before banking

### Outcomes of 339 referrals for elective semen cryopreservation

Of the 339 men requesting sperm cryostorage, 313 had cryostored semen samples. The remaining 26 men were either azoospermic (*n* = 22, 6.5 %) or had immotile spermatozoa (*n* = 4, 1.2 %). Disposition of cryopreserved sperm categories included continued storage (58 %), discarded (31 %), death (1 %), and use (4 %) (Table 3). Haematological neoplasms were the major cancers related to failed cryopreservation (46.2 %). Semen samples from patients with germ cell tumours (29.3 %) and haematological neoplasms (26.8 %) were the most abundant samples under current storage (Table 3).

**Table 3:** Sperm banking outcomes of each type of six cancers in each way of disposition

	Germ cell tumors	Hematological neoplasms	Head and neck cancers	Thoracic tumors	Abdominal tumors	Other tumors	Total
Ongoing storage	60(29.3 %)	55(26.8 %)	37(18.0 %)	8(3.9 %)	21(10.2 %)	24(11.7 %)	205
Destroyed	22(20.4 %)	28(25.9 %)	25(23.1 %)	6(5.6 %)	10(9.3 %)	17(15.7 %)	108
Used	2(15.4 %)	4(30.8 %)	4(30.8 %)	0(0 %)	2(15.4 %)	1(7.7 %)	13
Death	0(0 %)	2(50 %)	0(0 %)	0(0 %)	1(25 %)	1(25 %)	4
Unfrozen	6(23.1 %)	12(46.2 %)	5(19.2 %)	0(0 %)	1(3.8 %)	2(7.7 %)	26

Percentages within brackets indicate the proportion of each type tumor in each way of disposition of cryopreserved spermatozoa

### Outcome of ART with cryopreserved semen

We analysed the details of men opting cryopreserved spermatozoa and their reproductive outcomes. As of 31 December 2019, straws from 13 patients (3.8 %) had been used in 15 ART cycles (one patient received three cycles, including intracytoplasmic sperm injection [ICSI], in vitro fertilisation [IVF], intra-uterine insemination [IUI]). Conceptions were achieved in 66.7 % cases (10 out of 15), with 50 % (5 out of 10) pregnancies resulting in delivery and 20 % into spontaneous abortion (2 out of 10). Overall, 30 % (3 out of 10) were clinical pregnancies. Two couples failed to conceive, and one embryo was cryopreserved. The details of these cases are listed in Table 4.

**Table 4:** Details of men using cryopreserved sperm and their reproductive outcomes

	Age at banking (year)	Numbers of samples	Raw semen			Frozen-thawed semen			ART^a^	Reproductive outcome	
			Volume (mL)	Count (10^6^/mL)	Motility (%)	Count (10^6^/mL)	Motility (%)	Recovery rates (%)		Clinical pregnancies	Number of births
Testicular tumor	27	6	2.6	3.4	48	2.9	31	64.6	ICSI	live birth	1
Testicular tumor	27	6	6	14	49	10	22	45	ICSI	live birth	1
Leukemia	34	10	2.3	134	48	92	37	77	ICSI	live birth	1
Leukemia	28	4	3.9	9	47	3	30	63.8	ICSI	live birth	1
Lymphoma	37	8	4.3	42	68	28	33	48.5	ICSI/IVF/IUI	no pregnancy	0
Lymphoma	26	3	3.0	31	54	20	35	64.8	ICSI	clinical pregnancies	0
Nasopharyngeal Carcinoma	36	3	2.9	80	37	64	23	62.2	IVF	abortion	0
Nasopharyngeal Carcinoma	35	5	1.8	79	58	NS	NS	NS	IVF	live birth	1
Nasopharyngeal Carcinoma	41	4	3.1	108	52	NS	NS	NS	IVF	no pregnancy	0
Thyroid tumor	32	4	3.6	78	42	66	23	54.8	IVF	abortion	0
Colorectal cancer	46	4	1.6	56	39	43	23	59	ICSI	embryo cryopreservation	0
Gastric cancer	41	3	1.8	93	46	40	25	54.3	ICSI	clinical pregnancies	0
Hemangiosarcoma	28	6	2.5	26	28	16	11	39.3	ICSI	clinical pregnancies	0

Abbreviations: *ART* assisted reproduction technology; *ICSI* intracytoplasmic sperm injection; *IVF* In vitro fertilization; *IUI* intrauterine insemination

Reproductive outcomes of 13 patients who used their banked samples for 15 assisted reproductive cycles. One couple tried to conceive with three times of assisted reproduction; twelve couples tried to conceive with only one time

## Discussion

As cancer survival rate improves, more attention is being directed from issues of cancer treatment toward enhancing quality of life of cancer survivors. Cryopreservation of semen samples is a non-invasive procedure and the main treatment option for male cancer patients [18].

In our study, we demonstrated that male cancer patients in Sichuan, China, used sperm banking for fertility preservation. During the 10 years of our study, 339 male cancer patients attempted to preserve fertility; this is the largest reported population opting cryopreservation in China. The cancers were divided into six types, germ cell tumours, haematological neoplasms, head and neck cancers, thoracic tumours, abdominal tumours, and other tumours. We found that men with germ cell tumours (including testicular cancer and extragonadal germ cell tumours) had inferior pre- and post-cryopreserved sperm concentrations as compared to those with other types of cancers. However, upon separate comparison of testicular cancer, leukaemia, and lymphoma patients, we found that leukaemia patients had the lowest sperm concentrations. In this study, 46.2 % patients with leukaemia and lymphoma had no spermatozoa to freeze. Some reports have shown that leukaemia patients have an innate suppression of spermatogenesis through an unknown mechanism [19–21]. In practice, a few cancer patients visiting our unit give up sperm preservation owing to the cost. We concluded that the urgent treatment time for further treatment might be the reason for this result. A study showed that sperm total motile count (TMC) < 1 million was the second most prevalent category of sperm quality among leukaemia patients during the first visit for semen collection [22]. It is imperative to communicate with clinicians in a timely manner to ensure that leukaemia patients bank sperms before cancer treatment.

Some studies have reported that sperm count and motility are significantly lower in men with testicular tumours [19, 23–26]. Shankara-Narayana N et al. found that sperm output positively correlated with total testicular volume [26]. Moreover, another study reported that 50.0 % of patients presented with a decrease in sperm concentration after orchiectomy. Even before surgery, approximately half of patients with testicular germ cell tumours presented poor semen quality [27]. Therefore, there are several factors underlying semen quality decline in patients with testicular germ cell tumours, including orchiectomy, disruption of the blood-testis barrier, and endocrine derangements [28, 29].

Cancer patients who choose to use their cryopreserved sperms for fertility will have a good chance of experiencing fatherhood. Nevertheless, studies have reported that the rate of frozen semen usage varies between 7 and 30 %, and about 50 % of them are successful for in vitro fertilisation and intracytoplasmic sperm injection [30–33]. Over the course of 10 years, 3.8 % patients used their samples in our facility. The time elapsed between sperm freezing and follow-up is a fundamental factor that determines the utilisation rate. The number of patients requiring this service is sharply increasing year after year along with the number of patients who opt to use their samples in assisted conception [33]. Without this information, it is difficult to evaluate the importance of the results. Even if the observation time was 10 years, it is possible that the last recruited patients had a very short follow-up time for evaluation. The limitations of our study are the inability to follow-up couples who were naturally conceived and the lack of knowledge about the reason for sample discard. In the future, we will follow-up all cancer patients who join our cohort for sperm banking for their reproductive outcomes and investigate the reason for sample discard.

Considering the late development of fertility conservation programmes, there is a large gap between China and Europe or the USA. In 2005, the proportion of haematological and germ cell cancer patients between 15 and 19 years of age who opted cryopreservation was 92 and 90 %, respectively in France [34]. At present, there is no report on oncologists providing suggestions on fertility protection in China, but the sperm preservation of tumour patients in various sperm banks in China reflects the current situation of fertility protection to a certain extent. There were 130 male patients who underwent sperm cryopreservation before proceeding to gonadotoxic treatment at the Chinese University of Hong Kong [35]. A total of 97 Chinese male patients underwent sperm cryopreservation in the Hunan Human Sperm Bank from 2004 to 2015. These retrospective audits revealed 52 men with cancer who successfully banked spermatozoa in Zhejiang Human Sperm Bank between 2005 and 2013, 12 men in Shanghai Human Sperm Bank between 2003 and 2009, and 17 men in Jiangsu Human Sperm Bank between 2007 and 2014 [10]. A total of 145 male cancer patients underwent sperm cryopreservation in Beijing Human Sperm Bank [11]. There were 339 male patients with cancer opting fertility preservation option in our unit.

No practice guidelines advocate fertility preservation for cancer in China. One obstacle to sperm banking is inadequate communication between physicians and patients regarding the risk of post-treatment infertility [36]. Specialists in male reproduction or oncologists should discuss sperm preservation possibilities with cancer patients as early as possible. All oncological therapies, including surgical procedures, chemotherapy, and radiotherapy, pose some risk to fertility [4]. These adults can cryopreserve spermatozoa produced by masturbation. To date, sperm cryopreservation is impossible for prepubertal male patients, especially those who cannot provide semen. If collection of seminal spermatozoa is not possible, the testicular tissue containing spermatogonia stem cells can be obtained by biopsy. Exciting basic scientific study is underway to address unmet needs of fertility preservation that may expand fertility options for men in the future.

## Conclusions

In conclusion, our study supports the finding that sperm banking is an effective method of fertility preservation in cancer patients. Fertility preservation is very low among Chinese male patients with cancer. Therefore, reproductive physicians and oncologists are required to discuss fertility preservation techniques before gonadotoxic therapy in all male cancer patients. Sperm cryopreservation is a quick and effective technique for preserving fertility. The development of local clinical guidelines and organisation of conferences to promote fertility preservation should be encouraged.

## Data Availability

The datasets used and analyzed during the current study are available from the corresponding author on reasonable request.

## Abbreviations

ART: Assisted reproductive treatments
WHO: World health organization
SSCs: Spermatogonia stem cells
EGTC: Extragonadal germ cell tumours
ALL: Acute lymphoblastic leukaemia
AML: Acute myelogenous leukaemia
CML: Chronic myelocytic leukaemia
NHL: Non-hodgkin lymphoma
HL: Hodgkin lymphoma
NPC: Nasopharyngeal carcinoma
ICSI: Intracytoplasmic sperm injection
IVF: In vitro fertilization
IUI: Intrauterine insemination
